# Nanostructures for Achieving Selective Properties of a Thermophotovoltaic Emitter

**DOI:** 10.3390/nano11092443

**Published:** 2021-09-19

**Authors:** Lucie Šimonová, Milan Matějka, Alexandr Knápek, Tomáš Králík, Zuzana Pokorná, Filip Mika, Tomáš Fořt, Ondřej Man, Pavel Škarvada, Alexandr Otáhal, Pavel Čudek

**Affiliations:** 1 Department of Electrical and Electronic Technology, Faculty of Electrical Engineering and Communication, Brno University of Technology, Technická 3058/10, 616 00 Brno, Czech Republic; cudekp@feec.vutbr.cz; 2 Institute of Scientific Instruments of the Czech Academy of Sciences, Královopolská 147, 612 64 Brno, Czech Republic; mmatejka@isibrno.cz (M.M.); knapek@isibrno.cz (A.K.); kralik@isibrno.cz (T.K.); zuza@isibrno.cz (Z.P.); fumici@isibrno.cz (F.M.); fortt@isibrno.cz (T.F.); 3 Central European Institute of Technology, Brno University of Technology, Purkyňova 656/123, 612 00 Brno, Czech Republic; ondrej.man@ceitec.vutbr.cz; 4 Department of Physics, Faculty of Electrical Engineering and Communication, Brno University of Technology, Technická 3058/8, 616 00 Brno, Czech Republic; skarvada@feec.vutbr.cz; 5 Department of Microelectronics, Faculty of Electrical Engineering and Communication, Brno University of Technology, Technická 3058/10, 616 00 Brno, Czech Republic; alexandr.otahal@vutbr.cz

**Keywords:** thermophotovoltaics, selective emitters, nanostructures, reactive ion etching, emissivity, electron beam lithography

## Abstract

This paper focuses on the research and development of a suitable method for creating a selective emitter for the visible and near-infrared region to be able to work optimally together with silicon photovoltaic cells in a thermophotovoltaic system. The aim was to develop a new method to create very fine structures beyond the conventional standard (nanostructures), which will increase the emissivity of the base material for it to match the needs of a selective emitter for the VIS and NIR region. Available methods were used to create the nanostructures, from which we eliminated all unsuitable methods; for the selected method, we established the optimal procedure and parameters for their creation. The development of the emitter nanostructures included the necessary substrate pretreatments, where great emphasis was placed on material purity and surface roughness. Tungsten was purposely chosen as the main material for the formation of the nanostructures; we verified the effect of the formed structure on the resulting emissivity. This work presents a new method for the formation of nanostructures, which are not commonly formed in such fineness; by this, it opens the way to new possibilities for achieving the desired selectivity of the thermophotovoltaic emitter.

## 1. Introduction

Thermophotovoltaics belongs to the third generation of photovoltaics. The main goal of the third generation is to increase the efficiency of photovoltaic systems by using new methods. The advantage of thermophotovoltaics (TPV) is the ability to utilize both visible (VIS) and infrared (IR) regions of radiation. Photons of infrared radiation have very low energy and are therefore not capable of photovoltaic conversion because the frequency and energy of the photons decrease alongside with increasing wavelength. Therefore, in order to be able to use also low energy photons, the TPV system must be composed of several parts. The first one is the PV cell. Based on the cell used and the width of its forbidden band, the other parts of the system are selected, which include the filter and the emitter (also called absorber or radiator). When choosing a suitable PV cell, several things need to be considered: the width of the forbidden band, availability, toxicity of the materials the cell is made of, and the price. For TPV systems, either silicon (Si; *E_g_* = 1.12 eV) or germanium (Ge; *E_g_* = 0.66 eV) cells can be used, but also cells containing elements such as gallium (Ga), indium (In), arsenic (As), antimony (Sb), phosphorus (P)-GaAs (*E_g_* = 1.42 eV), and GaSb (*E_g_* = 0.72 eV), as well GaInAs, GaInAsSb, and InAsSbP and many others [1,2].

Depending on which PV cell is used, the parameters of the other two parts of the system are selected. One of them is the filter, which may or may not be part of the system. Therefore, to maximize the efficiency of the entire system, the filter used must meet the following basic conditions: minimum radiation absorption, high reflectivity for photons with energy (*E*) lower than the width of the forbidden band (*E_g_*) of the used PV cell back to the radiator, and maximum transmittance for photons with energies *E* > *E_g_* [3]. Instead of a filter, a reflective layer on the back of the PV cell can also be used to reflect unwanted photons back to the radiator [3,4]. Whether a filter is needed in the system is determined by the characteristics of the most important element of the system, which is the radiator.

The TPV radiator performs two basic functions in the system—that of an absorber and an emitter. Closer to the radiation source is the absorber side of the radiator, which is supposed to absorb as much of the incident radiation on the surface of the radiator as possible. The other side of the radiator, the one closer to the cell or filter, acts as an emitter of photons to be used in PV conversion. The basic characteristic of a proper radiator is the ability to emit maximum photons of energy *E* ≥ *E_g_* and minimum photons of energy *E* < *E_g_* [3]. A constant temperature is important for the proper function of the radiator [1,4]. The temperature is maintained in two ways: from the radiation source and by back-reflected photons, whose energy is very small for conversion but sufficient to maintain the temperature of the radiator. These photons can contribute to heating the radiator or can be used in further emission.

Radiators can be divided into two types according to their specific properties: grey and selective radiators. The main feature of a grey radiator is the same spectral emission for all wavelengths. It is therefore obvious that when using this radiator, a filter will also be needed for proper spectral management of the system [1,4]. Selective radiators, on the other hand, are characterized by selectivity, i.e., sensitivity to certain wavelengths [4,5,6].

Depending on the heat source used by the TPV system, it can be divided into three groups, which then indicate the actual area of application. The first is the “Solar TPV System”, where the sun is the source of radiation. These systems do not differ much from conventional PV systems in terms of appearance and/or use. The only difference is the need to focus the sun radiation using lenses (mirrors) to achieve a sufficient radiator temperature. The second group of TPV systems are so-called “Nuclear TPV Systems”, which are based on the decay of radioisotopes. The greatest use is expected to be in deep space where solar radiation is too small for conversion. The last group comprises “Controlled Combustion Systems”, where various forms of combustion (gases, solid fuels) are used as a heat source and the resulting heat is used for TPV conversion. This system operates on the principle of heat recovery, where heat can be produced for heating and at the same time this heat can be used for electricity generation. For the proper functioning of this radiator, its constant temperature is important, which should be 1200 ≤ *T_E_* ≤ 2000 K (900 °C or more) [1,4]. There is great interest in this technology, especially in areas without electricity, such as mountainous areas and northern states. The same interest is also shown by armies or hybrid vehicle manufacturers [1,4]. It is therefore possible to say that this design system can have a very wide commercial application compared to the first two variants, and we have therefore investigated this issue in more detail.

The first references to thermophotovoltaics began to appear around the 1950s. One of the first to address the issue of selective emitters was Robert E. Nelson, followed by others. Interest in the field of TPV declined after a while but picked up again at the turn of the 20th century for several reasons. The biggest of them is the noticeable progress in photovoltaics and the development of new technologies that have made other approaches possible. Of course, much of the recent development has been due to increased interest in the use of alternative energy sources [1,4]. There are several directions that this area of research has taken in recent years. The main differences are the materials used, their porosity, combinations, and the technologies used to treat them. Most of the selective emitters created in experiments around the world to date have shown enhanced emissivity in the infrared region. 

In our research, we wanted to achieve increased emissivity also in the visible spectrum and thus push the boundaries of TPV systems’ operations and uses, where silicon cells can be used in addition to germanium or gallium PV cells with much higher efficiency of the resulting TPV system.

In recent years, the development of TPVs has taken different paths. If we only focus on the area of using a tungsten substrate to produce a selective emitter, then in addition to micro and nanostructures, some of the research has also focused on thin films. A typical example is the use of a basic W substrate, W structures, and thin films of other materials. An interesting direction is the use of a nanostructured selective emitter consisting of two thin layers of Si_3_N_4_ silicon nitride and W in between. It is intended to increase the emissivity of the surface in conjunction with the concentration of solar radiation. The system has been tested for GaSb photovoltaic cells [7].

Theoretical analyses and simulations play an important role in the development of TPV systems. They focus, for example, on the use of thin films and cavity reflectors to enhance the performance of the TPV system [8,9]. Parametric studies are also key to understanding how changes in temperature and solar radiation affect the flow of electricity and heat [10]. Another important area is selective absorption and emission spectra, which have a major impact on the performance of the overall system. Systematic thermal analyses of TPV systems can help optimize the selective emitter to maximize the efficiency of the overall system. In [11], the combination of W-HfO_2_ materials for the selective emitter and GaSb photovoltaic cells is very interesting. These have been analyzed together with the structures. Simulations confirmed enhanced emissivity at low directional sensitivity [12]. With the help of simulations and calculations, further possibilities, optimizations, and directions for the development of TPV systems for different PV cells can be investigated very efficiently.

## 2. Materials

The main objective was to achieve the elimination of undesirable thermal effects on the PV cell while increasing the efficiency of TPV systems by using a specially developed selective emitter in the visible and near-infrared region of the spectrum. Based on the thermal requirements for the selective emitter, high temperature materials were used. The theoretical assumptions and the requirements imposed on the thermal and selective properties of the emitter were verified using experiments. Among the potentially suitable materials for the field of selective emitters, after several experiments, we took a closer look at the tungsten substrate.

### 2.1. Methods and Analyses

Different methods of substrate surface pretreatment and subsequent surface treatment were used to achieve the desired emitter selective properties. Based on the results of the methods used, we subsequently focused mainly on the use of lithography and reactive ion etching (RIE), through which we created very fine structures with the desired parameters in order to achieve an increase in the selective properties of the emitter. This technology is commonly used in various fields; however, due to the substrate used and the fineness of the structures, new techniques had to be developed to achieve these requirements. The aim was to develop a method, using the available means, to create very fine structures that are not commonly produced and thus achieve the required selectivity.

The structures or holes created act as cavity resonators. The resonance properties of the holes were first simulated and then experimentally verified. The created structures and the underlying substrate were continuously observed by microscopy and subjected to emissivity tests.

#### 2.1.1. Basic Principle of the Design of Nanostructures and Verification by Simulations

In designing and creating nanostructures for improving emissivity in the VIS and NIR spectra, we relied on the principle of waveguides, which are able to resonate at a specific wavelength based on their dimensions. We performed calculations of circular cavities that are intended to act as waveguides for them to be as close as possible to the needs of commercially available silicon PV cells, which cover mainly the VIS and possibly the NIR spectrum. Thus, we used as a basis the width of the forbidden band *E_g_* of a silicon PV cell, where *E_g_* = 1.11 eV. Based on this value, we could determine the required resonant wavelength *λ_nm_* [13]:

(1) $$\lambda_{nm}=\frac{h\cdot c}{E_{g}}=\frac{4.357\cdot 10^{-15}\cdot 2.998\cdot 10^{8}}{1.11}=1.117\;\mu m$$

where *h* is Planck’s constant [eV], *c* is the speed of light [m·s^−1^] and *E_g_* is the width of the forbidden band. From the calculated value of the resonance wavelength, we were already able to calculate the required diameter of the cylindrical cavity *D* [nm]. The calculation of the diameter of the cylindrical cavity [13] is derived from the formula:

(2) $$\lambda_{nm}=\frac{\pi D}{\rho_{nm}}\;or\;\frac{\pi D}{\rho_{nm}^{\prime }}$$

where *D* is the diameter of the cylindrical cavity, and *ρ_nm_* and *ρ’_nm_* denote the m-th zero point of the n-th Bessel function *J_m_*. The smallest possible values of these parameters give the cut-off wavelength *λ* [13]. Using Bessel functions, it is possible to solve a number of problems that have rotational or spherical symmetry. In optical systems such as the structures we desire, rotational symmetry is common. Thus, after derivation, we obtain the following formula for the cavity diameter [13]:

(3) $$D=\frac{\lambda_{nm}\cdot \rho }{\pi }=\frac{1.117\cdot 1.841}{\pi }=0.655\;\mu m\;$$

where *ρ* is a constant determined from the root of the first order Bessel function, and *π* is the Ludolph number. The constant *ρ* corresponds to the TE_11_ mode (transverse electric wave), which is the dominant mode in the cavity. Thus, the ideal dimensions of the structure should have a cavity diameter of 655 nm, a cavity depth of 440 nm, and a period of max 1 μm. The cavity depth was determined experimentally, based on the findings of available previous foreign experiments obtained using simulations.

**Figure 1:**
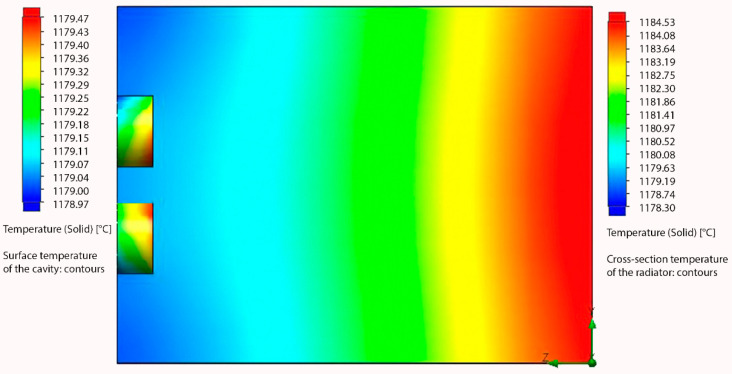
Simulated heating of a tungsten emitter with micro/nanostructure. Adapted from [15] with kind permission from Brno University of Technology.

Due to the financial requirements of the materials and processes used, we performed simulations before the actual physical formation of the structures to see how the desired nanostructure should work in real life in terms of resonance in the structure. The simulation was carried out using Solidworks 3D CAD software. For this purpose, we used the Solidworks Flow Simulation module to observe the heat propagation in the selective emitter. Flow Simulation works with the Finite Volume Method (FVM), which works on the principle of dividing a given region into a finite number of small volumes using a mesh [14]. For the purpose of the simulation, a 1 cm thick tungsten material was defined at a source temperature of 1200 °C, which corresponds to the operating temperature range of the radiator, as it is illustrated in Figure 1.

Based on the simulation, the heat propagation in the cavities compared to the surroundings could be seen, suggesting that the expected resonance inside the cavities is indeed occurring. Based on the calculation and simulation, we already physically formed the structures; the physical creation of the structures was preceded by the process of cutting the samples and surface treatments needed to achieve the desired results.

#### 2.1.2. Surface Treatment and Cutting of Samples

Among the options offered for cutting the tungsten substrate, mechanical cutting, laser cutting, and waterjet cutting were considered. When selecting the method, the time required, the availability of the technology, the efficiency of the design in relation to the material used, and many other factors were considered. Waterjet cutting proved to be the most suitable method. The method offers very high cutting accuracy even for hard and thick materials, regardless of the shape to be cut. The cutting itself is a highly efficient and environmentally-friendly process; the cut edge and surface do not need further processing as in other methods, and the cold cutting process does not cause any stress on the material, thus preserving the material’s material stability; the material is not thereby affected by physical and/or chemical processes either.

Three sets of 1 mm thick samples were cut from a 99.95% tungsten rolled sheet using an abrasive waterjet cutting system: square specimens of 20 mm × 20 mm, circular specimens of 3” in diameter, and square specimens of 10 mm × 10 mm. The first two sets were used for the basic experimental part and fine-tuning of new sample surface treatments and then the formation of nanostructures to find the optimal procedure. The third set of samples was used for re-verification of procedures developed by us and final measurements. 

After cutting the samples, we focused on achieving the finest possible surface roughness of the samples. Given the required parameters of the proposed nanostructure, the surface roughness of the samples is a key factor. Again, we used several methods to achieve the finest possible surface roughness. We repeatedly measured the roughness before and after the process using a non-contact profilometer (Keyence International, Mechelen, Belgium) and observed the changes on the sample surface using a microscope. The best results were obtained by mechanical lapping using diamond lapping pastes of different roughnesses. Lapping was performed in two stages, namely coarse lapping followed by fine lapping to remove any undesirable effects caused during the first lapping stage. This process was carried out in collaboration with the Thermo Fisher Scientific Company using professional lapping equipment.

#### 2.1.3. Surface Roughness Measurement with Profilometer

**Figure 2:**
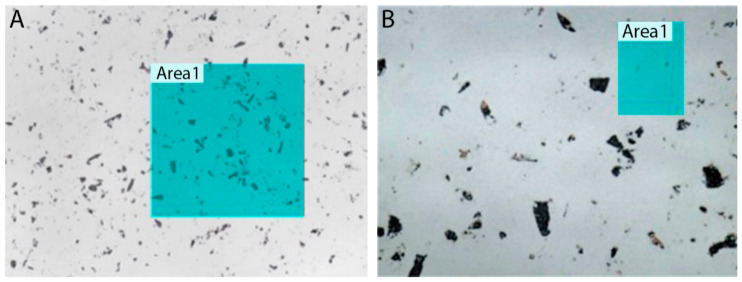
In-surface roughness (laser + optical microscope): (**A**) surface with larger unevenness (×50); (**B**) surface with small unevenness (×150). Adapted from [15] with kind permission of Brno University of Technology.

**Figure 3:**
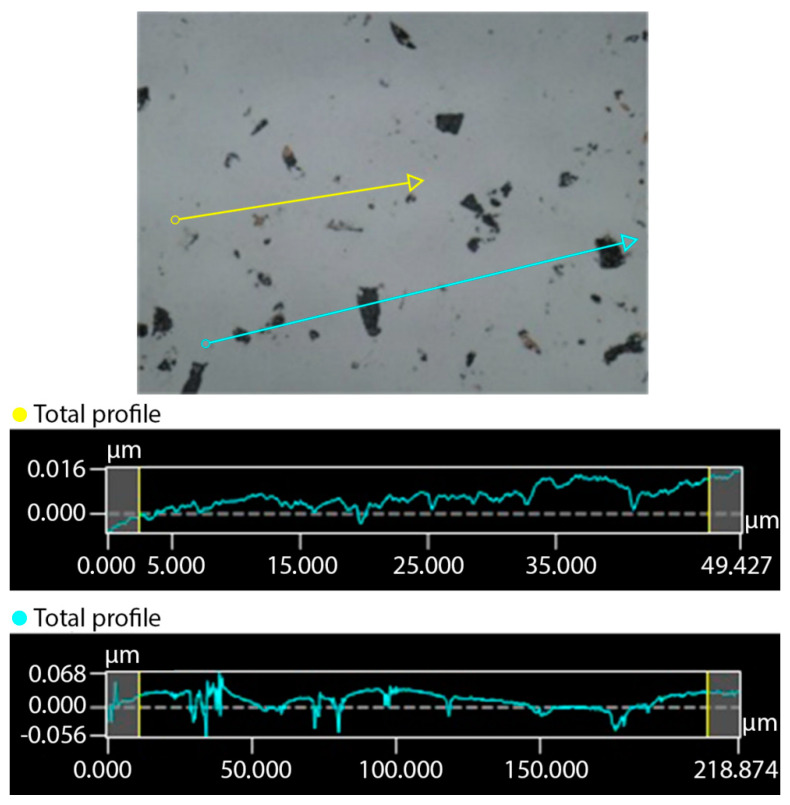
Roughness of the tungsten in-line sample: yellow line with small surface unevenness; blue line with large surface unevenness (×150). Adapted from [15] with kind permission of Brno University of Technology.

The samples were then subjected to both in-line and in-surface roughness measurements in order to obtain the most complete picture of the surface roughness. Looking at the samples under a Keyence VK-X Series 3D laser scanning confocal microscope (Keyence International, Mechelen, Belgium), it was apparent that the surface was not completely even. Therefore, for both the in-surface (Figure 2) and in-line (Figure 3) measurements, two distinct areas were always selected—one with a noticeably small amount of unevenness, and one with a significantly larger amount of unevenness. These unevennesses, as it was shown later, represented the remnants of corundum after polishing that had adhered to the substrate surface. For the measured in-surface roughnesses, we arrived at a range of *R_a_* = 8 nm for surfaces with small unevenness and *R_a_* = 23 nm for surfaces with larger unevenness. For the in-line roughnesses, we arrived at a range of *R_a_* = 3 nm for surfaces with small unevenness and *R_a_* = 34 nm for surfaces with larger unevenness. The original surface roughness of the samples before lapping was around 600 nm.

#### 2.1.4. Reactive Ion Etching

**Figure 4:**
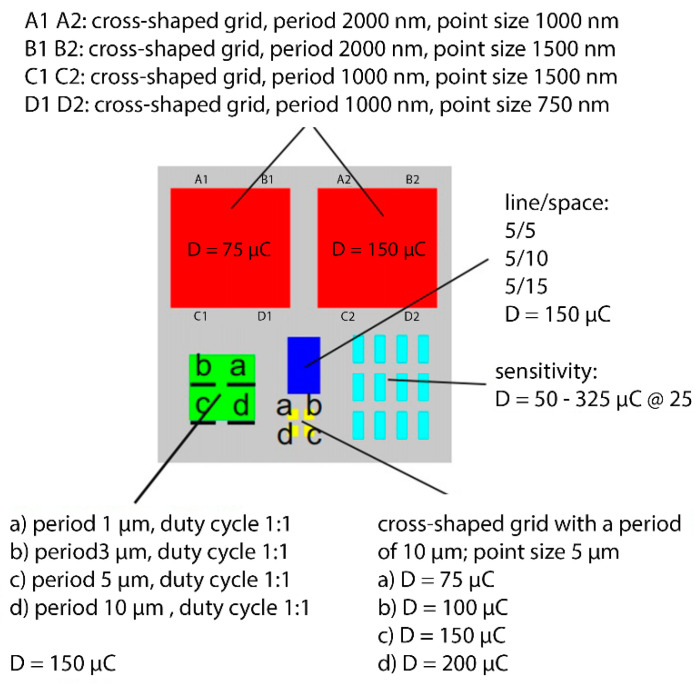
One of the mask motifs with multiple structures. Adapted from [15] with kind permission of Brno University of Technology.

The formation of structures using RIE was carried out with the support of the Institute of Scientific Instruments, Academy of Sciences, in Brno. Electron lithography and reactive ion etching techniques allow the recording of micro and nanostructures from different types of inorganic and organic layers or surfaces [16]. However, the sample used in the experiment, i.e., from rolled tungsten (containing large grains of material), is not exactly one of the standard types of sample on which lithographic operations are performed, such as recording the emission nanostructure. Therefore, it was necessary to design an optimal etching procedure of this material for the formation of nanostructures. We had to perform several test of recording of the pattern in resist type etching mask using electron lithography with subsequent plasmatic and RIE to find out the optimal process of fabrication of the desired pattern. The important role in designing a new fabrication process for forming designed nanostructures in the lapped tungsten substrate was played by selection of the type of electron resist used for etching mask, method and parameters of its deposition on samples (like soft-baking temperature and time), exposure parameters, and development parameters. The pattern motifs on the etching mask were chosen to include test and measurement structures, coarse lattice motifs, and the desired fine lattice nanostructure, so that we were able to gradually optimize the process of producing the structures (Figure 4) and eliminate unwanted effects on the substrate and the structure formed.

After recording the grid motifs of the etching mask by electron lithography, these structures were measured by AFM (atomic force microscopy; Nanopacific). The rationale for this was to be able to compare the loss of resist mask material during etching in RIE and to determine its masking properties, e.g., etching selectivity. Because we had no previous experience with etching the material of the tungsten (rolled) substrate, we designed and experimentally tested two types of RIE recipes. For the first experiment we used the process based on recipe-resist AZ2070 (deposition by centrifugal casting, 2500 rpm/30 s), drying (hot plate, 100 °C/90 s), exposure (UV, type e-flood, 151.2 mJ/cm^2^), developer (mif 726/150 s), measurement (contact profilometer), RIE (CHF3 + SF6, IPC power 3000 W/RIE power 100 W/120 s/60 °C), and measurement (contact profilometer). Despite adjustments to the etching time and RIE power, we did not achieve the desired results. The etching of tungsten surface was very slow—in 120 s and at 100 W power at RIE electrode and 3000 W IPC power, we reached only 100 nm according to the measurement on contact profilometer KLA-Tencor Alpha D-120 Stylus (KLA Instruments, Milpitas, California). For this recipe we observed a relatively rapid loss of mask material at a relatively low tungsten etching rate, so we decided not to continue with this etching recipe. For the next experiments we changed the RIE recipe for an SF6 + Ar-based process. Our assumption was that Ar ions would help enhance the etching reaction. The etching time for the experiment remained the same, but we reduced the RIE electrode power to 50 W. As a result of these changes, the etching process was quite different from the previous procedure. According to the measurement from contact profilometer, we reached a depth of etching of about 1200 nm. The etching was therefore much faster. We again observed a high rate of resist thickness loss and roughening of the etched surface due to Ar ion bombardment. We therefore retained the gas used but adjusted the RIE power, etching time, and number of repetitions at short time intervals so that the sample could thermally relax between the individual etching cycles. By this, we achieved a gradual etching without the undesirable effect of damaging the substrate and better selectivity of etching through the resist mask.

#### 2.1.5. SEM Observations

**Figure 5:**
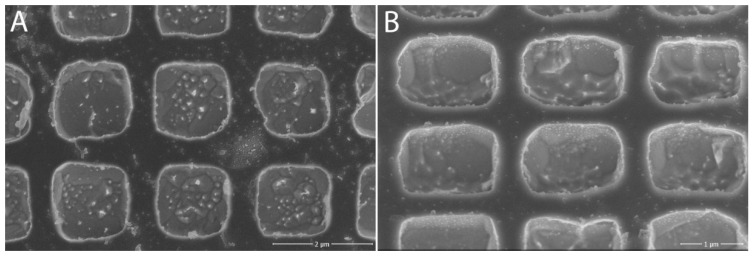
Lattice A2 under SEM microscope: (**A**) view of the structure; (**B**) at 45° inclination. Adapted from [15] with kind permission of Brno University of Technology.

The formed structures in the different fields with different coarseness of the structure were subjected to closer examination under the SEM microscope (Magellan, Thermo Fisher Scientific, Waltham, MA, USA). For the A2 lattice (Figure 4 and Figure 5, respectively), unevenness in the formed holes could be seen, which could be due to both the etching time and impurities on the surface of the tungsten chip. A closer look showed that by etching, we exposed the crystalline structure of the tungsten material itself.

**Figure 6:**
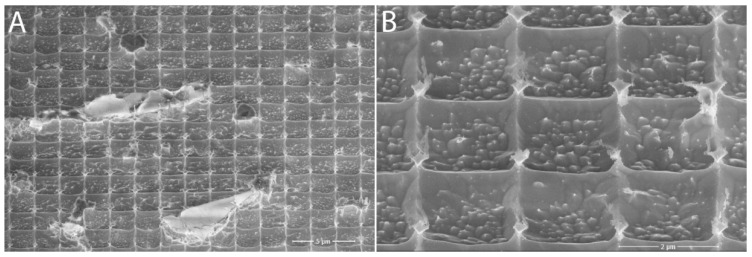
Lattice B2 under SEM microscope: (**A**) overall view including impurities; (**B**) closer view of the formed structure. Adapted from [15] with kind permission of Brno University of Technology.

In lattice B2 (Figure 6), the spacing between the holes was already almost negligible. Their shape was regular and again, on a closer look, the structure of the tungsten chip itself could be seen, which thanks to etching came out much more than the A2 lattices. The only drawback we encountered with all the structures we created was dirt on the surface of all of the samples, which could not be removed by normal sample surface cleaning procedures. This issue has been addressed in further research. 

**Figure 7:**
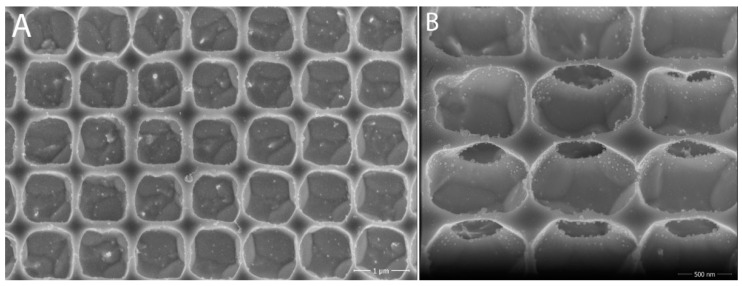
Lattice C2 under SEM microscope: (**A**) overall view; (**B**) zoomed view of irregularities and damage to the holes. Adapted from [15] with kind permission of Brno University of Technology.

For the C2 lattice, the regularity of the formed structure was quite regular at first glance, but a closer look showed that there was a slight rounding of the edges in some holes (Figure 7), which was probably due to the longer etching times in different steps. On closer inspection of the interior of each hole, it appeared at first that microlenses had formed (lens effect); however, when looking at the structure at a 45° inclination, we found that these were not microlenses, but that underetching had occurred in most holes. We also looked at the strange patterns at the bottom of the holes. After an initial assessment, we thought that these were crystallography-channeling. However, there was no noticeable difference at 1.5 keV and 10 keV energies, so they were likely to be a manifestation of morphology, with preferential etching of some crystallographic directions.

At first glance, it is possible to notice the similarities between our artificially created structures and naturally occurring photonic structures. Both use the principle of cavity period and cavity resonance for certain wavelengths. Naturally occurring photonic structures in some crystalline substances or animals acquire their optical properties precisely due to the periodic structure, which can be 1D, 2D, or 3D. A similar effect can be achieved with artificially created structures, the application of which can be wide.

### 2.2. The Resulting Nanostructure

**Figure 8:**
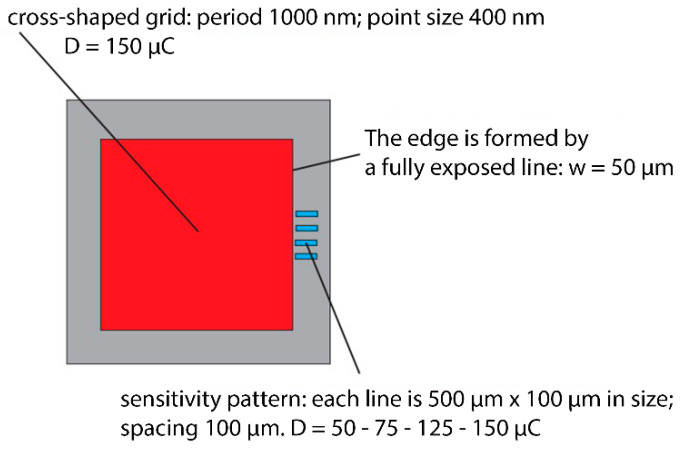
Main mask motif for the desired structure. Adapted from [15] with kind permission of Brno University of Technology.

The outcome of the above procedures was to achieve a process thanks to which we were able to create the desired unbroken structure over the entire surface of the sample, excluding the edges that remained structureless, for better manipulation. We used the similar process we developed on test samples for preparation of an etching mask, as well as the RIE etching recipe except for the resist, etching time, and number of repetitions of etching. Between the etching steps we monitored the depth of the etching in tungsten at the lines of the sensitivity pattern at the edge of the sample (see Figure 8).

For the final experiment we used the improved process based on recipe-resist 6200.13 CSAR (deposition by centrifugal casting, 4000 rpm/45 s), drying (hot plate, 150 °C/90 s), exposure (e-beam, 150 μC/cm^2^), developer (nAAc/190 s), measurement (contact profilometer), RIE (SF6/50 + Ar/40, IPC power 3000 W/RIE power 15 W/60 s/60 °C), plasma resist stripping (O_2_, IPC power 3000 W/RIE power 100 W/60 s), and measurement (contact profilometer).

**Figure 9:**
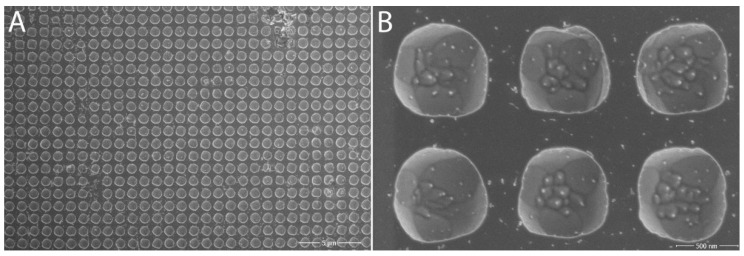
Resulting lattice on tungsten chip: (**A**) overall view; (**B**) detail of the structure of the formed cavities (50×). Adapted from [15] with kind permission of Brno University of Technology.

The resulting lattice achieved the desired parameters, i.e., cavity diameter of 655 nm, cavity depth of 440 nm, and period of max 1 μm. After fabrication, the structure on the sample was observed by the SEM microscope. As the images from SEM shows (see Figure 9) we were able to archive basically the desired structure in the tungsten. The actual dimensions corresponded to the desired ones; moreover, when viewed at a 45° angle, there was no underetching and/or distortion of the structure and holes, as was the case in the previous experiments. The edges of the holes were again rounded, but this was not an issue for us, as circular holes have a much greater effect for our purposes. As with the other experiments, the crystalline structure of the tungsten substrate was visible at the bottom of the cavities.

As with the previous structures, the main drawback here was the impurities that irregularly covered the tungsten sample and thus introduced considerable error into the entire process.

#### Measurements of Nanostructure Emissivity

On the resulting tungsten samples, emissivities were measured using the cryogenic method, which is designed to measure emissivity and absorption in highly reflective surfaces [17]. The measurements were carried out at the Institute of Scientific Instruments, Academy of Sciences. The measurements were always performed on a sample without structure and then with the resulting fine structure, which was previously created using RIE. A common sample size for these measurements is 40 mm in diameter, which corresponds to the requirements of the test chamber. In order to be able to measure emissivity even on our considerably small non-circular samples, a copper ring plate was used as a base carrier.

Since the tungsten samples are considerably small compared to the total surface of the carrier, different procedures had to be tried in order to make the measurements possible. The copper itself has an emissivity of up to 1% [18], but when stuck to the center of the tungsten sample carrier, the dark to black edges could introduce a large error into the measurement. One percent of 100% black area on the reflective surface of a sample with 1% absorption capacity will increase the overall absorption capacity of the sample to approximately 2%. It was necessary here to address the issue of eliminating parasitic effects when the sample is not over the entire surface of the Cu carrier. This factor has a large effect on the resulting absolute accuracy of the results.

After several adjustments and measurements at 240 K, the temperature at which emissivity is best measured [17], we then carried out reference measurements. We measured the emissivity of the carrier used in the range of 20–320 K. We then glued a tungsten sample to the center of the Cu carrier and taped its edges with Al tape. On the second piece of the carrier, we glued Al tape in the same way at the same spacing, but this time without the tungsten sample. Preparation of one sample before measurement took about 1 day, then for each measurement another day at least, sometimes more, depending on the results and necessary adjustments and/or technical problems.

**Figure 10:**
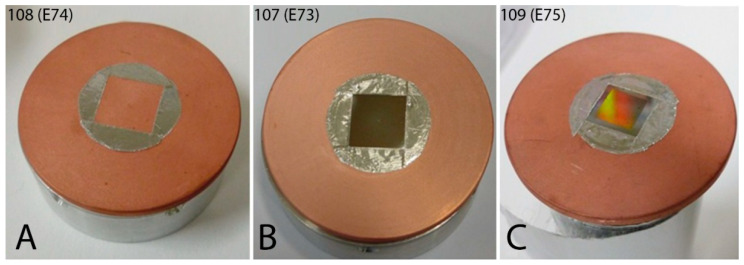
Samples for emissivity measurement and subsequent correlation: (**A**) Cu carrier with Al tape; (**B**) Cu carrier with pure tungsten sample and Al tape; (**C**) Cu carrier with tungsten sample with structure and Al tape. Adapted from [15] with kind permission of Brno University of Technology.

In the next step, we measured the emissivity of the Cu carrier itself in a given temperature range. Subsequently, we measured the emissivity of the Cu carrier with Al tape under the same conditions. Only after that we measured the carrier with a pure tungsten sample wrapped with Al tape and finally the carrier with a tungsten sample with structure, also wrapped with Al tape identically as in the previous cases. Since each of the materials introduces some intrinsic emissivity into the final measurement, which for us actually means an error, it was necessary to correlate all results to the tungsten sample without structure and then with structure (Figure 10). In this case, the Cu carrier, Al tape, and the area of pure tungsten formed the background for us so that we were able to determine the resulting emissivity only of the formed structure on the tungsten sample.

**Figure 11:**
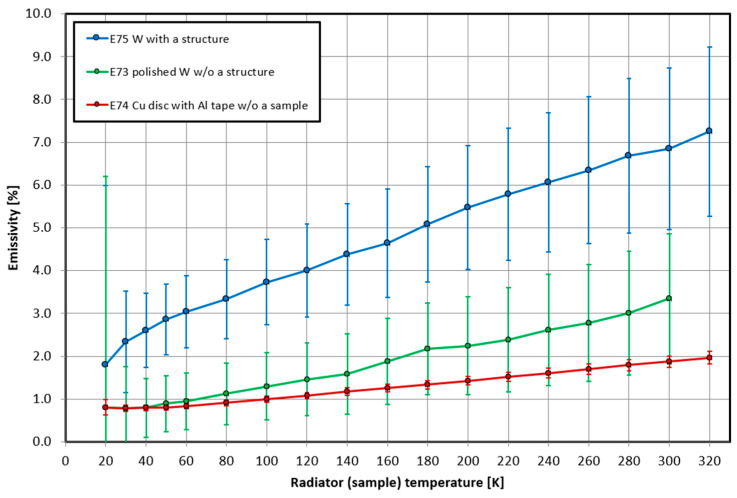
Resulting emissivity of tungsten sample with structure (**blue curve**) after correlation relative to the emissivity of the pure tungsten substrate (**green curve**) and the underlying copper carrier with aluminum tape (**red curve**). Adapted from [15] with kind permission of Brno University of Technology.

After performing the correlation, we obtained the emissivity of the tungsten sample with fine structure. The emissivity ranged from 1.8% to 7.25% depending on the temperature (Figure 11), i.e., 20 K–320 K. Using Wien’s displacement law, we could calculate the range of wavelengths that corresponded to the given temperatures at which the emissivity was measured. For temperatures of 20 K–320 K we were in the wavelength range of 144.90 μm–9.056 μm, which corresponded to the infrared region of the radiation.

Comparing the emissivity of the pure tungsten sample and the emissivity of the formed structure, a significant increase in emissivity was obvious, which is highly desirable for use in selective emitter applications for TPV. In order to obtain a better idea of the emissivity under different conditions, it would be advisable to perform this experiment several more times in the future. The only disadvantage of this method is that it is designed for measurements only up to room temperature, and it is not possible to use it to verify the emissivity at higher temperatures. Even so, it is clear from the diagram that the emissivity of the structure increased with increasing temperature, which suggests that it will behave very well at higher temperatures. Thus, the emissivity should be more noticeable also in the visible region, which is crucial for this experiment.

## 3. Results and Discussion

The main aim of the work was to use commonly available technology and develop a new method specifically for the creation of nanostructures in order to increase the emissivity of the samples to put their selective properties in line with the needs of silicon PV cells. The TPV systems researched so far in the world mainly specialized in the use of VF cells made of materials such as arsenite–gallium, which achieved higher yields only in the IR region, whereas silicon ones were more suitable for the VIS and NIR region. This was the area we focused upon. The dimensions of the structure were calculated based on the width of the forbidden band of the silicon PV cell and using Bessel functions that are commonly used for resonator calculations. 

Prior to the actual creation of the fine structures, we performed a simulation using 3D CAD Solidworks where we verified whether—when the desired structures were created—this structure or individual cavities would work as a resonator to support the selective properties of the emitter.

For the creation of very fine structures, the surface roughness is a key component of the substrate used. For this reason, we focused on surface roughness treatment in the first stage. Among the possible methods, based on experiments, we chose mechanical lapping as the most effective one, which was carried out in two phases—coarse and then fine—to achieve the best results. After selecting the optimal procedure, we reached roughnesses of 3 to 34 nm from the original 600 nm. 

For the creation of the nanostructures, we used many performed experiments as a basis and chose one method, which according to our results was the most effective, namely RIE technology. In the course of designing a new procedure and optimizing the process to create the resulting nanostructure with a cavity diameter of 655 nm, cavity depth of 440 nm, and max period of 1 µm, we proceeded to achieve increased emitter selectivity in the VIS and NIR regions. During all the experiments, we tried to remove unwanted effects such as underetching that arose during the procedure optimization. Therefore, we proceeded from the coarsest motifs to the finest ones. In this way, we were able to eliminate many imperfections that might otherwise have occurred during the formation of the final nanostructure. The main shortcoming in the entire process of sample preparation and subsequent formation of fine structures was the impurities on the surface of all samples. These impurities could not be cleaned by conventional methods. Thus, their influence was also evident in the formation of the resulting structures. This topic is being further addressed in subsequent research, as it is a major issue.

For the resulting nanostructure, we performed emissivity testing to determine if there was an improvement in the emitter selective properties, which is a very key factor for this work. Since the surface of the tungsten sample was highly reflective, we could not use conventional methods for emissivity measurements. For this purpose, it was possible to use the cryogenic method, which is designed to measure emissivity from 20 K to 320 K, corresponding to wavelengths in the infrared region. Due to the size of the sample and the possible introduction of errors into the measurements from the surface edges of the sample, which were darker, we arrived at a very positive result after several measurements based on correcting the results for focusing on the structure. The emissivity of the lapped tungsten substrate ranged from 0.8% to about 3%, whereas the emissivity of the tungsten sample with structure, or the emissivity of the structure itself, ranged from about 2% to 7%. Thus, we can say that the desired increase in emissivity due to the fine structure created by our method was indeed confirmed. In this experiment, we were limited by the temperatures at which we could measure the emissivity, which means that for temperatures around 900 K and above, it was not possible to directly measure it within this technology. However, from the curve of emissivity increase as a function of temperature, it can be expected that even at higher temperatures, i.e., even in the visible region of the spectrum, the emissivity of the structure will increase. In the future, it would be useful to simulate the expected evolution of emissivity at higher temperatures using computational programs.
